# Hyperammonemic Encephalopathy due to Valproic Acid and Topiramate Interaction

**DOI:** 10.1155/2014/410403

**Published:** 2014-07-17

**Authors:** Jennifer D. Twilla, Andrew S. Pierce

**Affiliations:** ^1^Methodist University Hospital, 1265 Union Avenue Memphis, TN 38104, USA; ^2^Department of Clinical Pharmacy, University of Tennessee Health Science Center, Memphis, TN 38163, USA; ^3^Department of Medicine, University of Tennessee Health Science Center, Memphis, TN 38163, USA

## Abstract

Valproic acid-induced hyperammonemic encephalopathy is a rare yet serious adverse drug reaction. Medication interactions such a valproic acid and topiramate can precipitate an event. We present the case of a 52-year-old female that presented with acute mental status change and hypersomnolence due to hyperammonemia caused by a valproic acid derivative. The patient improved after withdrawal of the offending medications and treatment with lactulose. Clinicians should remain hypervigilant in monitoring for valproic acid-induced hyperammonemic encephalopathy and risk factors such as polypharmacy.

## 1. Introduction

Valproic acid (VPA) is considered one of the first line treatment agents for psychiatric conditions such as bipolar disorder [1]. Despite the widespread use of this medication in psychiatric disorders, little literature has been published reviewing the association with VPA and hyperammonemic encephalopathy in psychiatric patients. Although common in the neurology literature, the first report of hyperammonemic encephalopathy associated with VPA use in psychiatric patients was in 1995 by Settle Jr. [2]. In 2012, a review by Chopra et al. reported that only 30 cases of valproate-induced hyperammonemic encephalopathy (VHE) along with 5 cases from their institution (total 35 cases) had been discussed in the literature [3]. Even though the development of VHE seems rare in this patient population, the literature addressing the presenting symptoms, risk factors, and treatment for VHE has been well documented.

Along with potential electroencephalography (EEG) findings, VHE is commonly characterized by lethargy, vomiting, cognitive slowing, focal neurological deficits, and decreasing levels of consciousness ranging from drowsiness to coma in patients on VPA treatment [3, 4]. Among the prevailing risk factors for this condition, the most widely accepted are urea cycle disorders, carnitine deficiency, and polypharmacy [5–8]. Common medications that may contribute to the development of VHE when used concurrently are phenytoin, phenobarbital, and topiramate.

## 2. Case

A 52-year-old female was brought from a care home to the hospital for altered mental status. Patient was nonverbal on arrival to hospital; therefore, history of events was given by case manager. Four days prior to admission she visited her psychiatrist where her medications were adjusted. According to the case manager from the care home, the patient was started on clonazepam 1 mg daily. Divalproex sodium 500 mg twice daily was restarted and quetiapine was increased from 100 mg to 150 mg in the evening. Following these adjustments, the patient was noted to have increased somnolence. Her past medical history included schizoaffective disorder, essential tremor, migraines, seizure disorder, cerebrovascular accident, and chronic obstructive pulmonary disease. Home medications were as follows: primidone 200 mg twice daily, topiramate 50 mg every morning and 100 mg every evening, paroxetine 30 mg daily, gabapentin 300 mg twice daily, quetiapine 50 mg every morning and 100 mg every evening, clonazepam 1 mg daily, and divalproex sodium 500 mg twice daily.

Due to her altered mental status and increasing somnolence, several laboratory and diagnostic tests were performed. On admission, vital signs were stable; patient was normotensive and afebrile; heart and respiratory rate were normal. Initially, infection was ruled out given that she had no evidence of leukocytosis, vital signs were normal, and blood, urine, and cerebrospinal fluid cultures were all negative. Urine drug screen and alcohol detection tests were negative except for barbiturates (patient on primidone). Liver and renal function tests were within normal limits. Initial computerized tomography (CT) of the brain was normal. Ammonia level was elevated at 132 mcmol/L. Valproic acid level on admission was 120 mcg/mL (reference range: 50–150 mcg/mL). Further studies to aid in elucidating the etiology of the patients change in status were ordered. This included an EEG, which showed moderate to severe slowing and no seizure activity. Findings were deemed to be consistent with metabolic encephalopathy.

Given laboratory abnormalities and patient presentation, all psychotropic medications were held on admission. The patient was started on lactulose 10 gm per nasogastric tube every four hours as needed to titrate to 3 bowel movements/day for encephalopathy, heparin 5000 units subcutaneous every 8 hours for venous thromboembolism prophylaxis, and continuous intravenous fluids. After 2 days of hospitalization, the patient became more responsive but was not back to baseline. On day 3 of hospitalization, patient developed a temperature of 38.6°C. A chest X-ray was performed and revealed a focal infiltrate in left lung. At that time, she was started on moxifloxacin for presumed community acquired pneumonia versus aspiration. With only mild improvement in patient responsiveness, decision was made to do a lumbar puncture which was negative. On day 6, a neurologist that had previously seen the patient was consulted. She noted that patient's mental status had improved, but that she was agitated and psychosis appeared worse. Per neurology, primidone, paroxetine, and quetiapine were restarted along with continued treatment of pneumonia with moxifloxacin to finish out course of 14 days. Upon day 10 of hospitalization, patient was noted to be back at baseline and ready for discharge once cleared by mobile crisis team. Patient was discharged in stable condition to a psychiatric unit. It was determined by internal medicine team as well as neurologist that acute encephalopathy was likely due to hyperammonemia induced by reinitiation of valproic acid and sedating effects of medications.

## 3. Discussion

Hyperammonemia is a known side effect of administration of VPA and its derivatives. Raja and Azzoni assessed adult patients admitted to a psychiatric unit receiving a mood stabilizer such as VPA and found that 51.2% of patients receiving VPA had asymptomatic hyperammonemia [9]. While the prevalence of asymptomatic hyperammonemia in this study is high, it is important to note that VHE is rare and often hard to distinguish from the development of acute psychosis. Per package labeling recommendations, there is no evidence for routine screening of ammonia levels in patients receiving VPA [10]. However, if a patient presents with unexplained lethargy and vomiting or changes in mental status then an ammonia level should be measured. Symptoms may vary from mild to severe changes in cognitive and behavioral function including acute mental status change, lethargy, cognitive slowing, increased seizure frequency, and death [2, 11, 12]. The diagnosis of VHE can be established by ruling out other causes of impaired consciousness along with laboratory (VPA level, ammonia level) and diagnostic studies (EEG). However, the notion that there is a correlation between symptoms and VPA daily dose or serum concentrations has been somewhat controversial. Upon review of previously published reports, there appears to be no discernable relationship between development of VHE and daily doses nor serum VPA levels [3, 4, 11, 13].

In VHE, the main pathophysiological mechanism involves the inhibition of an enzyme involved in the urea cycle with subsequent development of hyperammonemia [4]. Ensuing CNS toxicity due to the hyperammonemia is thought to be mediated by excessive activation of the NMDA type of glutamate receptors [14]. This sequence of events leads to the development of VHE. Urea cycle disorders, carnitine deficiency, and polypharmacy provide a framework for the above mechanisms to occur more readily. Previous studies have shown that topiramate inhibits both urea cycle and glutamate synthetase activity which are two of the primary mechanisms involved in the development of VHE [8, 15]. The polypharmacy our patient experienced (received both topiramate and divalproex sodium) was thought to be the cause of her acute mental status change. While there is no consensus regarding treatment other than withdrawing VPA therapy, several approaches have been used successfully in clinical practice [3]. These include supplementation with carnitine, lactulose, or neomycin and protein restriction [3]. For asymptomatic elevations in ammonia levels that persist, package labeling recommends to consider discontinuation of the medication [10].

## 4. Conclusion

We present the case of a 52-year-old female that developed presumed VHE after the addition of VPA to her home medication of topiramate. Given her high normal VPA level, elevated ammonia, presenting symptoms, and EEG findings, the diagnosis of VHE was made. This can be further substantiated by the Naranjo probability scale for adverse drug reactions (ADR) [16]. The score for this patient is a 6 which places her in the probable ADR category. This case highlights the need for clinician awareness regarding medication interactions and potential adverse effects. While VPA is commonly used in neurology and psychiatric patient populations, clinicians treating psychiatric patients may not be as familiar with all of the interacting antiepileptic medications. Since VHE if often hard to distinguish from acute psychosis, it is important to consider all aspects of the patient case including medication interactions.

## References

[1] Hirshfeld R, Bowden CL, Gitlin MJ (2002). American Psychiatric Association: practice guideline for the treatment of patients with bipolar disorder (revision). *The American Journal of Psychiatry*.

[2] Settle EC (1995). Valproic acid-associated encephalopathy with coma. *The American Journal of Psychiatry*.

[3] Chopra A, Kolla BP, Mansukhani MP, Netzel P, Frye MA (2012). Valproate-induced hyperammonemic encephalopathy: an update on risk factors, clinical correlates and management. *General Hospital Psychiatry*.

[4] Segura-Bruna N, Rodriguez-Campello A, Puente V, Roquer J (2006). Valproate-induced hyperammonemic encephalopathy. *Acta Neurologica Scandinavica*.

[5] Raskind JY, El-Chaar GM (2000). The role of carnitine supplementation during valproic acid therapy. *Annals of Pharmacotherapy*.

[6] Zaccara G, Paganini M, Campostrini R (1985). Effect of associated antiepleptic treatment on valproate-induced hyperammonemia. *Therapeutic Drug Monitoring*.

[7] Warter JM, Marescaux C, Brandt C (1983). Sodium valproate associated with phenobarbital: effects on ammonia metabolism in humans. *Epilepsia*.

[8] Hamer HM, Knake S, Schomburg U, Rosenow F (2000). Valproate-induced hyperammonemic encephalopathy in the presence of topiramate. *Neurology*.

[9] Raja M, Azzoni A (2002). Valproate-induced hyperammonaemia. *Journal of Clinical Psychopharmacology*.

[10] Depakote (2014). *Package Insert*.

[11] Verrotti A, Trotta D, Morgese G, Chiarelli F (2002). Valproate-induced hyperammonemic encephalopathy. *Metabolic Brain Disease*.

[12] Wadzinski J, Franks R, Roane D, Bayard M (2007). Valproate-associated hyperammonemic encephalopathy. *Journal of the American Board of Family Medicine*.

[13] Carr RB, Shrewsbury K (2007). Hyperammonemia due to valproic acid in the psychiatric setting. *The American Journal of Psychiatry*.

[14] Felipo V, Butterworth RF (2002). Neurochemistry of ammonia. *Neurochemistry International*.

[15] Deutsch SI, Burket JA, Rosse RB (2009). Valproate-induced hyperammonemic encephalopathy and normal liver functions: possible synergism with topiramate. *Clinical Neuropharmacology*.

[16] Naranjo CA, Busto U, Sellers EM (1981). A method for estimating the probability of adverse drug reactions. *Clinical Pharmacology and Therapeutics*.

